# Learners’ Perspectives on Interprofessional Simulation and Co‐Debriefing: An Exploratory Mixed‐Methods Study

**DOI:** 10.1155/jonm/3787497

**Published:** 2026-02-26

**Authors:** José Luis Díaz-Agea, Álvaro Ros-Romero, César Leal-Costa, Gabriel Segura-López, Pedro Simón Cayuela-Fuentes, José Antonio Vera-Pérez, Juan Manuel Cánovas-Pallarés, Manuel Piñero-Zapata, César Cinesi-Gómez, María Gracia Adánez-Martínez, María José Pujalte-Jesús

**Affiliations:** ^1^ Faculty of Nursing, University of Murcia, Murcia, Spain, um.es; ^2^ Faculty of Nursing, Catholic University of Murcia, Murcia, Spain, ucam.edu; ^3^ Faculty of Medicine, Catholic University of Murcia, Murcia, Spain, ucam.edu; ^4^ Faculty of Medicine, University of Murciam, Emergency Department of the ‘Virgen de la Arrixaca’ Hospital, IMIB, Murcia, Spain; ^5^ Health Emergencies Service 061, Region de Murcia, Spain

**Keywords:** co-debriefing, emergency, experiential learning, healthcare teamwork, interprofessional simulation, medical and nursing education, simulation training

## Abstract

**Introduction:**

Interprofessional simulation enhances both technical and nontechnical skills among healthcare professionals, improving their clinical practice. Co‐debriefing, where two facilitators lead debriefing sessions, is a common approach in these trainings. This study aims to assess educational aspects of interprofessional simulation and co‐debriefing in postgraduate medical and emergency nursing students.

**Methods:**

A descriptive cross‐sectional study with a mixed‐methods approach was conducted among postgraduate students (*n* = 46). A mixed‐methods design was used, combining quantitative questionnaire data with qualitative content analysis of open‐ended responses, allowing integration of numerical trends with in‐depth learner perspectives. A valid and reliable ad hoc questionnaire was designed, and qualitative content analysis was used to examine participants’ free‐text responses.

**Results:**

Students’ perceptions were evaluated across six dimensions: applicability, satisfaction, motivation, safe environment, organization, and co‐debriefing. Significant differences were found between medical and nursing students in all dimensions except applicability and organization. Overall, students rated interdisciplinary sessions and co‐debriefing positively, though nurses highlighted areas for improvement, such as icebreaker activities, more dynamic simulations, and equal representation of both professions.

**Conclusions:**

While interprofessional simulation and co‐debriefing were well received, participants—especially nurses—identified areas for enhancement to ensure a more balanced and engaging learning experience. By integrating quantitative outcomes with qualitative insights, the study highlights when co‐debriefing adds value in interprofessional simulation and when single‐facilitator approaches may be sufficient.

## 1. Introduction

Interprofessional simulation‐based education (SBE) is a globally recognized approach to improving communication, teamwork, and patient‐centered care among healthcare professionals [1–4]. International guidelines, including those from the World Health Organization (WHO) [5], emphasize the relevance of interprofessional education in enhancing collaboration and patient safety across healthcare systems worldwide. In addition to these global recommendations, several specialized scientific societies have contributed substantially to defining best practices in SBE. The Society for Simulation in Healthcare (SSH) and the International Nursing Association for Clinical Simulation and Learning (INACSL) have developed internationally recognized standards that provide guidance on the design, implementation, and evaluation of high‐quality simulation programs, thereby complementing and operationalizing the broad directives proposed by the WHO.

In Spain, nurses and physicians usually pursue separate undergraduate education pathways and typically come together for the first time during clinical internships, where effective collaboration becomes essential for delivering high‐quality patient care. This mirrors international trends aimed at enhancing healthcare quality through improved interdisciplinary cooperation. In this context, the Healthcare Simulation Standards of Best Practice–Simulation‐Enhanced Interprofessional Education (Sim‐IPE) published by INACSL offer a structured, evidence‐based framework to support the development of interprofessional simulation activities [6]. These standards are further complemented by INACSL guidelines on simulation design [7] and debriefing processes [8], which highlight key elements such as psychological safety, structured facilitation, and reflective learning. Recent contributions published in Clinical Simulation in Nursing reinforce the value of interprofessional simulation, demonstrating its impact on shared professional identity, collaborative behaviors, and learner engagement across health professions [9–11]. Integrating these international standards and empirical findings strengthens the theoretical foundation of interprofessional simulation and situates the present study within current global best practices.

Interprofessional SBE has gained prominence in higher education institutions, facilitating the development of essential skills within a collaborative, realistic environment [1, 2]. WHO [5] defines interprofessional education as a learning experience in which students from multiple professions engage in mutual learning, fostering collaborative skills to enhance healthcare delivery. Despite the increasing use of co‐debriefing in interprofessional SBE, a notable gap remains in empirical evidence regarding its effectiveness, best practices, and impact on learner outcomes [12].

Interprofessional simulation not only creates safe learning environments and enhances the realism of clinical training but also strengthens communication, fosters teamwork, improves understanding of roles and scopes of practice, and ultimately contributes to higher patient safety and quality of care [3].

Human factors, including poor communication, inadequate teamwork, fatigue, and stress, are recognized as contributors to errors in healthcare and pose a significant risk to patient safety [13–15]. In this context, interprofessional education aims to address these challenges by developing competencies in environments that simulate real‐world clinical scenarios, thereby improving communication, teamwork, and ultimately, clinical outcomes [4, 16].

Simulation‐based interprofessional team training programs have been shown to strengthen collaboration, enhance teamwork skills, and directly impact the quality of care and patient safety [17]. In these programs, structured debriefing plays a crucial role, as a trained facilitator guides it and promotes reflection, team‐based decision‐making, and continuous improvement in patient safety [18, 19].

Previous studies on interprofessional education between nurses and doctors [20, 21] have highlighted its benefits, such as increased understanding of other professions, professional empowerment, and overall satisfaction. However, challenges remain, including resource management, facilitation, and differing professional perspectives [22, 23].

Although single‐facilitator debriefing remains common, co‐debriefing—where two facilitators from different professions lead the simulation—has increasingly become popular [24]. Co‐debriefing presents unique challenges, including ensuring that facilitators agree on learning goals and preventing any one profession from dominating the discussion [25]. There remains debate over the necessity of co‐debriefing in all contexts, with some questioning whether it always enhances learning [26].

This study aimed to evaluate the perceptions of postgraduate medical and emergency nursing students regarding interprofessional simulation, with a specific focus on co‐debriefing, and to compare the results between these professional groups. Secondary objectives included identifying strengths and weaknesses of the co‐debriefing approach and formulating practice recommendations.

## 2. Materials and Methods

### 2.1. Design

This study employed an exploratory mixed‐methods, cross‐sectional design aimed at examining postgraduate medical and nursing students’ experiences during interprofessional simulation training. The exploratory nature of the study is explicitly acknowledged, as the purpose was to obtain an initial understanding of learners’ perceptions of interprofessional simulation and co‐debriefing in a specific educational context, rather than to test hypotheses with a powered sample. The design integrated quantitative data collected through a structured questionnaire with qualitative data derived from open‐ended written responses, allowing for a comprehensive description of participants’ experiences and perceived learning. Data collection took place from March to June 2022 at the Catholic University of Murcia (UCAM), Spain, as part of a joint training program for Medicine and Emergency Nursing students.

### 2.2. Participants and Sampling

A convenience sample was used, comprising the total population of postgraduate students enrolled in the Medicine (*n* = 27) and Emergency Nursing (*n* = 27) programs who were invited to attend the interprofessional simulation sessions during the 2021–2022 academic year (*N* = 54). Of these, 46 students (23 Medicine and 23 Emergency Nursing) completed the questionnaire and provided free‐text responses, yielding a response rate of 85% and constituting the final study sample. The remaining students either did not attend at least one simulation session or did not complete the questionnaire and were therefore excluded from the analysis.

Although the sample size is modest, it represents the entire cohort eligible for participation, which is appropriate for descriptive and exploratory mixed‐methods studies in SBE. Given the division into two equal professional groups, we acknowledge that the quantitative component has limited statistical power; therefore, the findings should be interpreted as preliminary and hypothesis‐generating. All participants met the inclusion criteria of enrollment in their respective postgraduate programs, attendance at the scheduled simulation sessions, and provision of informed consent. Participants were recruited via email invitations and in‐class announcements, with follow‐up reminders to optimize participation.

The inclusion criteria were: (a) being currently enrolled postgraduate students in the Medicine or Emergency Nursing program at the Catholic University of Murcia (UCAM) during the 2021–2022 academic year; (b) attending and actively participating in both scheduled interprofessional simulation sessions as part of the collaborative training curriculum; and (c) providing written informed consent for participation, which includes permission for video recording and data collection.

The exclusion criteria included: (a) incomplete participation—students who missed any scheduled simulation sessions or did not engage in all three clinical cases; (b) prior experience—students who had previously taken part in a similar interprofessional simulation to prevent bias from prior exposure; refusal of consent—students who declined to sign the informed consent form or did not agree to video recording; and (c) noncompletion of surveys—failure to complete the ISCAQ questionnaire or provide qualitative feedback after the sessions.

### 2.3. Simulation Sessions and Debriefing Structure

To facilitate the execution of the simulation sessions, participants were organized into groups of 12–13 students, maintaining a balanced representation of both professions (6–7 per discipline). Within each group, students were further subdivided into smaller interprofessional teams of 3–4 participants for the execution of the simulation cases. This organizational structure ensured active participation, promoted interprofessional interaction, and allowed learners to rotate roles across the different scenarios. The subdivision of groups is presented in this section, as it pertains specifically to the logistics and implementation of the simulation activities.

**FIGURE 1:**
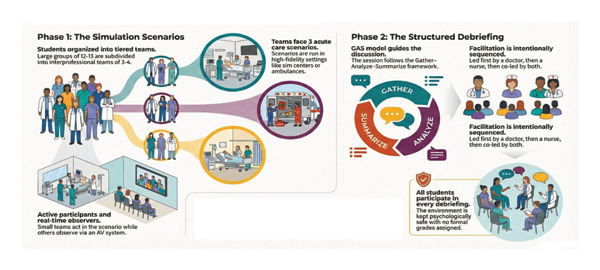
Simulation sessions and debriefing structure.

Each group completed two simulation sessions, each involving three clinical cases (see Figure 1). To facilitate the implementation of the sessions, participants were organized into groups of 12–13 students, maintaining a balanced representation of both professions. For each scenario, an interprofessional team of 3–4 students participated actively, while the remaining learners observed the simulation in real time through the audiovisual system in an adjacent room. All scenarios were conducted either in UCAM’s high‐fidelity simulation center or in a prehospital environment using a fully equipped ambulance to enhance realism.

The scenarios represented acute care situations requiring prompt assessment and teamwork, including two hospital‐based emergency cases and one prehospital case. Each scenario lasted 12–15 min, followed by a structured debriefing. The debriefing sequence followed a sequential approach: the first debriefing was facilitated by a medical educator with over 5 years of simulation experience, the second by an experienced nurse educator, and the third was co‐led by both facilitators to promote interprofessional dialog. This progression allowed learners to first reflect from their own professional perspective before engaging in a joint interprofessional analysis. Debriefings lasted 20–25 min for single‐facilitator sessions and 30–35 min for the co‐debriefing.

All students, including observers, participated in every debriefing session. The debriefings followed the GAS (Gather–Analyze–Summarize) model [27], a widely used approach in SBE. Facilitators did not maintain ongoing direct teaching relationships with the participants, which helped reduce familiarity bias, although some had previously interacted with students during general simulation workshops.

To minimize potential bias in performance evaluation, all scenarios were standardized using identical scripts, learning objectives, and debriefing frameworks. Participants rotated through different roles to reduce the effect of prior familiarity with cases. No formal grades or summative assessments were assigned, ensuring that the learning environment remained formative and psychologically safe.

### 2.4. Data Collection

#### 2.4.1. Quantitative Measures

Data collection took place immediately after the final simulation session. It involved gathering sociodemographic and professional information (such as profession, age, and prior simulation experience) and administering the Interprofessional Simulation and Co‐debriefing Assessment Questionnaire (ISCAQ), a specially designed, self‐administered paper form. To protect participant anonymity, questionnaires were completed without personal identifiers and placed in sealed envelopes.

The ISCAQ is an ad hoc instrument developed in Spanish specifically for this study. It was not adapted or translated from an existing tool. Its construction followed an extensive review of the literature on interprofessional simulation, co‐debriefing, psychological safety, and learner satisfaction [22, 25]. An initial pool of items was drafted based on these domains and subsequently refined through expert consensus.

To ensure content validity, the questionnaire underwent systematic evaluation by a panel of six university‐affiliated simulation debriefers, each with at least 2 years of experience in simulation‐based facilitation. These experts independently assessed item clarity, relevance, coherence, and conceptual adequacy. Items that did not meet agreement thresholds were revised or removed. The final set of items achieved a content validity index (CVI) of 1.0, indicating complete expert agreement regarding their adequacy. A separate student pretest was not conducted, as the questionnaire was intentionally designed to be applied to the full study population within this exploratory framework.

The ISCAQ comprises six dimensions that assess different aspects of the interprofessional simulation experience:

Applicability evaluates the perceived usefulness of the training (scores 3–15).

Satisfaction with interprofessional learning measures participants’ perceived benefit and knowledge gained (scores 2–10).

Motivation assesses learners’ interest in future simulation activities (scores 3–15).

Safe environment examines psychological safety, comfort, and trust during the sessions (scores 5–25).

Organization refers to the structure, clarity, and facilitation of the sessions (scores 3–15).

Co‐debriefing evaluates the effectiveness of two facilitators working jointly during debriefing (scores 7–35).

The total questionnaire score ranges from 23 to 115, providing a comprehensive measure of learners’ perceptions of interprofessional simulation.

Internal consistency was assessed using Cronbach’s alpha (*α*). Reliability values were acceptable to excellent across the six dimensions: Applicability (*α* = 0.702), Satisfaction (*α* = 0.741), Motivation (*α* = 0.760), Safe environment (*α* = 0.862), Organization (*α* = 0.827), and Co‐debriefing (*α* = 0.871). The total scale demonstrated outstanding reliability (*α* = 0.942), supporting the instrument’s robustness in this initial phase of psychometric evaluation. Detailed items and structure are presented in Table S1 (Supporting information).

#### 2.4.2. Qualitative Measures

For the qualitative component, participants provided anonymous written responses to open‐ended questions addressing their perceptions of co‐debriefing, facilitator characteristics, and differences between single‐facilitator and dual‐facilitator debriefings. These questions were developed through expert consensus and aligned with established qualitative inquiry frameworks in SBE [19, 28]. Data collection continued until all eligible participants had contributed, and theme saturation was reached during the analysis phase, as no new categories or subcategories emerged from the data.

The open‐ended questions were intentionally framed to support an exploratory and reflective inquiry, progressing from broad experiential prompts to more focused comparative reflections on debriefing formats and facilitator roles. This structured sequencing was designed to facilitate depth of response while avoiding prescriptive or evaluative language. Participants were encouraged to justify their perspectives, and the presence of both positive and critical responses suggests that the framing did not constrain or bias the expression of diverse viewpoints.

The opinions of participants regarding co‐briefing were collected at the end of each group’s session. Anonymously and individually, participants were encouraged to freely express their opinions on the suitability of co‐debriefing and the contrast perceived with single‐debriefer debriefing. The participants were asked to answer the following question: How did you feel during the interprofessional simulation? Did you find any differences between cases directed by a single debriefer as compared to one directed by two? If so, what are these differences? Do you believe that a good debriefer is characterized by being an expert on the subject or by being skilled in the facilitation tasks? Do you believe it is necessary to have two co‐debriefers when a simulation is planned that involves doctors and nurses? Why? Do you believe that co‐debriefers should always be representatives of the professions that train with simulation? Why?

### 2.5. Data Analysis

Quantitative data were processed with SPSS v21. Descriptive statistics (means, standard deviations, frequencies, and percentages) were computed for all variables. Normality of the ISCAQ dimension scores was examined using the Kolmogorov–Smirnov test and visual inspection of distributions, and homoscedasticity was assessed with Levene’s test. Although the sample size was modest, the distributions did not show substantial deviations from normality, so Student’s *t*‐tests were retained to compare Medicine and Emergency Nursing students, with a significance level set at *p* < 0.05. In addition, Cohen’s *d* effect sizes and their 95% confidence intervals were calculated for between‐group differences in each ISCAQ dimension to quantify the magnitude and precision of the effects. Years of professional experience and previous exposure to interprofessional simulation were examined descriptively and explored as potential covariates; as no clear confounding patterns were identified, formal multivariable models were not fitted.

For the qualitative data, an inductive content analysis approach was employed, drawing on established methodological principles described by Taylor, Bogdan, and DeVault [29], as well as other foundational authors in qualitative inquiry [30, 31]. The analytic process began with data familiarization, during which two researchers independently read and re‐read all participant responses to obtain an in‐depth understanding of the content. This was followed by open coding, generating initial codes closely grounded in the participants’ narratives, in alignment with the inductive strategies proposed by Taylor et al.

Coding proceeded through a process of constant comparison, where emerging codes were systematically examined within and across transcripts to identify patterns, contrasts, and conceptual relationships. Codes were subsequently synthesized into higher‐order categories and subcategories through iterative refinement. Throughout the process, the researchers maintained analytic memos and an audit trail, ensuring transparency and methodological rigor consistent with Lincoln and Guba’s criteria for trustworthiness—credibility, dependability, confirmability, and transferability [32].

Discrepancies between coders were resolved through structured consensus meetings. Theme saturation, defined as the point at which no new codes or conceptual insights emerged from the data, was reached, reinforcing the completeness and robustness of the findings. Although formal inter‐rater reliability statistics were not calculated, the trustworthiness of the analysis was enhanced through researcher triangulation, reflexivity, and adherence to the COREQ (Consolidated Criteria for Reporting Qualitative Research) guidelines [33].

### 2.6. Ethical Considerations

The data collected were handled anonymously and used solely for this study, adhering to current data confidentiality laws and basic ethical standards. All participants provided informed consent to take part and agreed to be recorded on video for educational or research purposes. Contributions were labeled with an alphanumeric code (Participant number (N) + professional category (M for Medical or N for Nursing) + gender (M for male or F for female); for example, 1 MF indicates the first female medical student and 5NM indicates the fifth male nursing student). The study received approval from the institutional ethics committee (Ref. Number: CE012107).

## 3. Results

### 3.1. General Results

Out of 54 postgraduate students (*N* = 54), 46 completed the questionnaire and provided free‐text responses, resulting in an 85% response rate. Most participants were women (80.4%), while men made up 19.6%. The average age was 28.59 years (SD = 5.5). The sample was almost evenly divided between medical doctors (47.8%) and nurses (52.2%). Regarding professional background, 69.6% of participants were employed at the time, with an average of 5.07 years of professional experience (SD = 3.8). Only 23.9% had previous exposure to interprofessional simulation, whereas 76.1% had never participated in such training before.

### 3.2. Quantitative Results

Survey results were organized and examined to reveal differences in responses between doctors and nurses (Table 1). Overall, participants gave high scores across all sections of the questionnaire. Nonetheless, there were notable statistical differences between the groups, particularly in satisfaction, motivation, perceived psychological safety, and the co‐debriefing assessment. Doctors consistently rated these aspects more favorably than nurses, with the largest gap in perceptions of a secure learning environment. These results suggest potential variations in the experience of interprofessional simulation across disciplines, highlighting areas for improvement in training methods. In addition to *p* values, effect sizes were calculated for all between‐group differences to quantify their magnitude beyond statistical significance. For the dimensions showing significant differences (Satisfaction, Motivation, Safe environment, and Co‐debriefing), Cohen’s *d* values ranged from moderate to large (*d* = 0.73–1.24), with 95% confidence intervals excluding zero (0.13–1.48 to 0.61–1.87), confirming these differences are educationally meaningful.

**TABLE 1:** ISCAQ questionnaire results.

Dimensions	Total		Profession	Mean	SD	Student’s *t* test	Sig. (*p*)	Cohen’s *d*	95% CI
	Mean	SD							
Applicability	12.63	1.57	Nurses	12.37	1.58	1.158	0.253	0.34	[(−0.24)–0.92]
			Doctors	12.90	1.54				

Satisfaction	8.83	1.40	Nurses	8.29	1.62	2.994	0.005	0.87	[0.27–1.48]
			Doctors	9.40	0.79				

Motivation	12.52	2.24	Nurses	11.66	2.53	2.994	0.005	0.88	[0.27–1.48]
			Doctors	13.45	1.40				

Safe environment	20.72	3.93	Nurses	18.75	4.29	4.255	0.000	1.24	[0.61–1.87]
			Doctors	22.86	1.90				

Organization	12.83	2.05	Nurses	12.37	2.46	1.587	0.113	0.47	[0.11–1.06]
			Doctors	13.31	1.35				

Co‐debriefing	29.09	4.81	Nurses	27.50	5.29	2.468	0.016	0.73	[0.13–1.33]
			Doctors	30.81	3.58				

### 3.3. Qualitative Results

When organizing the data from participants’ responses, four main categories and nine subcategories were identified (Table 2).

**TABLE 2:** Categories and subcategories of qualitative study.

Emotions during the Interprofessional simulation	Positive	Comfort
		Usefulness
	Negative	Teamwork
		Insecurity
		Dissatisfaction Feeling of not being “sufficiently valued”, by nursing Lack of previous knowledge before the sessions

Positive aspects of Co‐debriefing	Realism	Promotes communication between students
		Health care experience of the professionals
	Enriching	Support between facilitators
		Contributing greater knowledge

Aspects that could be improved in Co‐debriefing	Development of the session	Avoid contradictions between the facilitators
		More invigorating and attractive sessions
		Prior ice‐breaking sessions
	Inequality/Disequilibrium	Grant both professions with the same importance
		Decrease the dissatisfaction of the nurses

The best assessed facilitator skills according to the students	Facilitation skills	Favors learning
		Favors a safe environment
		Promotes the interest of the student
	Expert on the subject	Provides answers with scientific rigor
	Both	Possesses the knowledge needed and can effectively communicate it

#### 3.3.1. Category 1: Emotions of the Students During the Interprofessional Simulation Sessions

In Category 1, there was an apparent saturation of data regarding positive feelings. Most students expressed positive emotions during the sessions. In this category, three concepts stood out to the students and accounted for the majority of comments. Overall, they emphasized the feeling of comfort, highlighted teamwork as one of the most positive aspects of the interprofessional sessions, and underscored the usefulness of these sessions for handling similar situations in healthcare practice with greater confidence and skills.

*“Comfortable, in a favorable environment in which all opinions are considered” (3NF) (…) “I felt good, comfortable, and with a good rapport with my classmates” (8MM) (…) “real life is working as a team, and each plays an essential function, so that taking part in interdisciplinary teams helps us come close to reality” (11MF).*

On the other hand, about one‐third of the participants reported experiencing negative feelings or emotions at some point during the sessions. Among these negative emotions, we can highlight students’ insecurity regarding their knowledge or the environment.

*“Out of place, somewhat uncomfortable due to the lack of trust with the rest of the peers” 7MF (…) “I felt a bit lost at the start of the simulation due to the lack of communication and working with people with whom I had not worked with previously (…)” 37MF.*

However, the most common negative feeling in the participants’ testimonies was the sense of inequality perceived by nurses compared to doctors, along with the feeling of not being sufficiently valued.

*“Nervous and somewhat uncomfortable, as I felt that the role of the doctors had more importance” (27NF) (…) “sometimes they listened to me, and other times not, and you feel frustrated” (35NF) (…) “On many occasions, they left us nurses in the background. Little intention to communicate” (44NF).*

#### 3.3.2. Category 2: Positive Aspects of Co‐Debriefing

One of the most underlined aspects was the realism contributed by the co‐debriefers to the sessions. Besides sharing their knowledge, experiences, and skills, communication was also encouraged between the two professions, fostering an environment of debate that closely resembled a real clinical setting.

*“More real when both doctors and nurses participated” (43MF).*

Another positive aspect was the enriching nature of the co‐debriefing, not only because of the value gained from the experiences of various debriefers, as previously mentioned but also because of the support they provide to each other and the beneficial complementarity of their knowledge and skills for the students.

*“Two opinions always provide more, even more so if they are different specialties (nursing and medicine). Greater knowledge, greater debate, greater enrichment” (26MM).*

#### 3.3.3. Category 3: Aspects That Could Be Improved in Co‐Debriefing

Similar to the previous category, this section focuses on objective aspects rather than emotional ones that students believed could be improved based on their experience with co‐debriefing.

Regarding the development of the sessions, some students mentioned the lack of dynamism in their questionnaires, as the session was led by two debriefers instead of just one.

*“The sessions with two debriefers tend to be too long and repetitive” (14MF).*

*“(…) sometimes two (facilitators) become very tedious” (14MF).*

Additionally, they mentioned the appearance of some inconsistencies in the information provided by each debriefer during certain sessions, which created a barrier to students’ understanding of the learning objectives.

*“(…) there was little communication between the facilitators. (…). I felt that in the end it was as if there was only one facilitator” (33NM).*

However, the area that could be improved, which was most often mentioned during the content analysis, was related to the feeling of inferiority or perceived inequality among nursing students.

*“(…) the same attention only to medicine was always shown” (28NF) (…) “the same value should be given to both professions, so that both doctors and nurses intervene” (31NF).*

#### 3.3.4. Category 4: The Best Assessed Facilitator Skills According to the Students

The highest‐rated debriefer skills among students were the ability to communicate facilitation talents.

*“(the facilitator) must be an expert, but overall, he or she must be a good educator, with the ability to transmit information in a manner that is simple, understandable, and dynamic” (29MF).*

On the other hand, a small percentage of students emphasized being an expert on the subject taught as an essential aspect of a debriefer.

*“I believe that one must know the subject to be worked on in depth, as this can ease the information in a manner that is more fluid and can also help address the questions or doubts that emerge” (33NM).*

Others emphasized the need to possess both skills: the ability to communicate effectively and extensive knowledge, to be a good debriefer.

## 4. Discussion

The analysis of questionnaire data confirmed significant differences in how doctors and nurses perceive interprofessional simulation. These differences were evident across all dimensions except “Applicability” and “Organization,” with the most significant disparity observed in perceptions of a safe learning environment.

Our findings align with previous studies, such as Díaz et al. [22], which reported that while joint training was highly valued, nurses tended to adopt a more critical perspective than doctors. Feelings of frustration among nurses were linked to a perceived lack of recognition of their contributions during simulated scenarios. Similarly, in our qualitative analysis, many students expressed positive feelings such as teamwork and usefulness, while nurses were more likely to report feelings of insecurity, undervaluation, and inequality. These challenges are consistent with previous literature, which identifies hierarchy, organizational factors, personal traits, and patient responsibility as barriers to effective interprofessional education [34–36]. However, some studies contradict these findings, suggesting that interprofessional training can mitigate perceived hierarchy [37].

Beyond hierarchy, various obstacles and perceptions influence the willingness of medical and nursing students to engage in interprofessional education. Not all students demonstrated satisfactory levels of effective and safe communication in individual scenarios. Students’ willingness to participate in interprofessional education is shaped by their attitudes, perceptions, and barriers associated with the nursing profession [23]. The perceived inequality expressed by nursing students may be partially explained by traditional role distributions and culturally embedded hierarchies between professions, which can be reproduced even within simulated learning environments. Additionally, aspects of simulation design—such as task allocation, leadership roles within scenarios, or facilitator emphasis during debriefing—may unintentionally reinforce these dynamics, shaping how nursing students experience participation and recognition.

The differences observed between medical and nursing students can be interpreted through established theoretical frameworks related to professional identity, hierarchy, and psychological safety [38]. Edmondson’s concept of psychological safety [39, 40] provides a useful lens to understand why nursing students reported lower perceptions of a safe learning environment, particularly in situations where hierarchical dynamics were salient. Feelings of being undervalued or less heard, as expressed by nursing participants, are consistent with the presence of hierarchical gradients that can inhibit speaking up and participation in both clinical and educational settings. In this sense, interprofessional simulation and debriefing not only function as educational strategies but also as social spaces where professional identities and power relations are enacted and negotiated. The findings suggest that, unless explicitly addressed, these dynamics may persist even in simulated environments, influencing learners’ engagement and perceptions of equity within the team.

Cultural differences also significantly influence the willingness of physicians and nurses to participate in interprofessional education. Studies indicate that interprofessional ACLS courses are particularly beneficial for nurses; however, significant differences exist between medical and nursing students. While nurses tend to take a collective approach to patient care, physicians often assume individual responsibility for their clinical duties. This cultural distinction impacts their willingness to engage in interprofessional education, with nurses showing greater enthusiasm than doctors [41].

One of the most important aspects of interprofessional education that contributes to frustration among nurses is the influence of professional social identity. A strong attachment to one’s profession can reduce the effectiveness of interprofessional SBE. The significant personal value placed on one’s professional group often dominates and limits opportunities for collaborative work in interprofessional teams. This phenomenon was also seen in broader experiences of IP‐SBE. The feeling of belonging to an “in‐group” can be a powerful force, sometimes acting as a barrier, but also potentially serving as a tool to support the goals of IP‐SBE. Future IP‐SBE sessions might focus on developing a shared “in‐group” identity based on factors beyond professional training, which could better fulfill the aims of interprofessional education [9].

From a nursing leadership and management perspective, these findings have direct implications for everyday clinical practice. Structured debriefing practices, whether in simulation or real clinical settings, can serve as powerful tools for nurse leaders to foster psychological safety and reduce hierarchical barriers within interprofessional teams. For example, applying debriefing principles after critical incidents, complex shifts, or interdisciplinary handovers may encourage nurses to voice concerns, share clinical reasoning, and participate more actively in decision‐making processes. By intentionally modeling inclusive facilitation strategies and promoting equitable participation, nurse leaders can translate the lessons learned from interprofessional simulation into daily practices such as shift handovers, multidisciplinary meetings, and quality improvement discussions, ultimately contributing to safer and more collaborative care environments.

Regarding motivation, all participants rated their experience highly, although doctors gave higher scores than nurses. Free‐text responses showed that both groups appreciated the realism of clinical cases and their relevance to real‐world practice. However, some students—mainly nurses—felt that the sessions lacked energy and stressed the importance of fair participation in both simulated scenarios and debriefing sessions.

Barriers to implementing interprofessional simulation training also include perceived hierarchical structures within healthcare. These hierarchies hinder student socialization due to ingrained attitudes and professional stereotypes acquired during their training. The existence of professional hierarchies limits collaborative learning and reduces opportunities for practical application [42].

A key challenge identified was the lack of initial rapport and psychological safety among participants. Since students had not interacted before, the absence of structured ice‐breaking activities affected their comfort levels. This was especially evident among nurses, who rated the safe environment dimension lower than doctors. Prior research supports these findings, highlighting difficulties in communication and role satisfaction among nurses in interprofessional simulation settings [43]. Psychological safety is widely acknowledged as crucial for effective simulation‐based learning [38, 44–46]. Recent studies suggest that self‐directed simulation [20, 47] can create a more supportive learning environment by encouraging team synergy and collaborative learning. This approach may also enhance communication and trust within interprofessional teams [45].

Besides social barriers, logistical challenges remain a major concern. Coordinating schedules is one of the biggest hurdles, along with instructor training, role clarification, and program alignment. These logistical issues complicate the implementation of interprofessional education and must be addressed to ensure its success [48]. Based on our institutional experience, the successful implementation of interprofessional simulation programs can be facilitated by early coordination between academic departments, securing recurring time slots in all participating curricula, cross‐training facilitators in both medical and nursing perspectives, and creating integrated simulation calendars well in advance.

Regarding co‐debriefing, participants appreciated having two facilitators because it promoted discussion and reflection across both professions, enhancing the learning experience. However, some students mentioned inconsistencies in the facilitators’ communication, which sometimes confused. A small group of participants also felt that having two debriefers led to longer, less engaging sessions. The literature suggests that a well‐structured and coordinated approach is crucial for maximizing the effectiveness of co‐debriefing [24, 49]. Our findings suggest that co‐debriefing is most advantageous in complex, multidisciplinary scenarios where learning objectives require perspectives from multiple professions, and when learners have limited prior exposure to interprofessional practice. In simpler cases or with highly experienced participants, a single skilled facilitator may suffice, ensuring efficiency without compromising learning outcomes.

Despite these challenges, having two debriefers was mainly seen as beneficial. Their complementary expertise enhanced interdisciplinary learning, underscoring the value of diverse perspectives in interprofessional simulation. Moving forward, improving facilitation techniques and encouraging equal participation could further strengthen the educational value of these sessions.

In interpreting the quantitative findings, it is important to acknowledge that the exclusively positive phrasing of items may have introduced a potential ceiling effect and that no pilot testing or factor‐analytic procedures were conducted due to the study’s exploratory mixed‐methods design and the limited sample size. These constraints are consistent with the early development stage of the ISCAQ and with the study’s aim to obtain an initial, descriptive understanding of learners’ perceptions; however, future research will benefit from larger samples, pilot refinement, and structural validation to further strengthen the instrument’s psychometric properties.

### 4.1. Limitations

This study had some limitations. The ISCAQ questionnaire, despite its valid and reliable indices, requires additional testing to achieve robust psychometric properties. The research was conducted within a local context at a Spanish university, which restricts its external validity. Multicenter studies might be necessary to enhance this validity. A pilot pretest of the ISCAQ with students was not performed. Instead, content validation by experts ensured clarity and adequacy of the items. Future studies should include pilot testing with independent samples and advanced psychometric validation.

Future validation steps for the ISCAQ will include multicenter data collection with a larger, more diverse sample; exploratory and confirmatory factor analysis to assess construct validity; and evaluation of convergent and discriminant validity through comparison with established instruments for measuring interprofessional education outcomes.

Although the qualitative component of this study was intentionally designed to capture learners’ subjective experiences of the interprofessional debriefing structure rather than to evaluate facilitator performance, we acknowledge that the inclusion of a validated instrument could have provided a complementary perspective. Future studies may therefore benefit from incorporating established tools such as the Debriefing Assessment for Simulation in Healthcare (DASH) [50], which is aligned with international debriefing standards (SSH, INACSL) and offers a structured, validated approach to assessing the effectiveness of facilitator behaviors and debriefing practices.

Using a convenience sample may have introduced selection bias, as participants who attended the sessions might have inherently more favorable attitudes toward interprofessional learning. Differences in demographic characteristics and prior professional experience, which were not fully controlled for, could also partly explain the perception differences observed between medical and nursing students.

Although meaningful qualitative insights were obtained, the absence of member checking should be acknowledged, as the anonymous and written nature of data collection precluded participant feedback on interpretations. In addition, the researchers’ backgrounds in nursing, medicine, and SBE may have influenced data interpretation. To mitigate this, independent coding, consensus discussions, reflexive memoing, and an audit trail were employed.

This study is limited by its modest sample size and exploratory design, which reduces the statistical power and the stability of effect size estimates. Potential confounders, such as years of professional experience and prior exposure to interprofessional simulation, were explored descriptively; however, no formal multivariable adjustment was performed, and residual confounding cannot be ruled out.

## 5. Conclusions

Satisfaction with interprofessional simulation varied between doctors and nurses. Despite these differences, both groups recognized the applicability of the cases to daily clinical practice and valued the realism of the presented cases. Motivation was high in both groups and was closely linked to the fidelity of the scenarios, which effectively mirrored real‐world healthcare settings.

Several areas for improvement were identified, particularly by nursing participants, including the need for greater session dynamism, equal recognition of both professions, and the establishment of a psychologically safe environment that promotes teamwork and open communication. Structured ice‐breaking activities and balanced participation in both scenarios and debriefings were also recommended to improve comfort and trust among participants.

Co‐debriefing was generally well received, as it enriched discussions by incorporating diverse perspectives, fostering interdisciplinary dialog, and providing multiple role models for learners. However, some participants perceived them as occasionally too long or inconsistent, suggesting the need for better coordination between facilitators.

At our institutions, these results will inform the integration of co‐facilitated debriefing in complex interprofessional scenarios, the systematic use of structured icebreakers to build rapport, and enhanced facilitator training to ensure consistency and equal representation of both professions in future course designs.

Co‐debriefing appears to be particularly beneficial in complex, high‐acuity interprofessional simulations that require explicit role negotiation and integration of multiple professional perspectives. In contrast, single‐facilitator debriefing may be sufficient for scenarios with clearly defined objectives and limited interprofessional interaction, offering a more efficient use of educational resources without compromising learning outcomes.

Implication for practice: This study suggests that interprofessional simulation can be enhanced by ensuring balanced representation of professions among facilitators, incorporating structured icebreakers to build rapport, and improving coordination between co‐debriefers to avoid redundancy and misalignment.

Recommendation for future research: Future studies should adopt multi‐center designs to increase external validity, explore strategies for fostering a shared interprofessional identity, and evaluate whether more dynamic and interactive simulation formats improve learning outcomes.

## Author Contributions

Conceptualization, José Luis Díaz‐Agea, César Leal‐Costa, Pedro Simón Cayuela‐Fuentes, César Cinesi‐Gómez, María Gracia Adánez‐Martínez, and María José Pujalte‐Jesús; data curation, José Luis Díaz‐Agea, Álvaro Ros‐Romero, César Leal‐Costa, and María José Pujalte‐Jesús; formal analysis, José Luis Díaz‐Agea, César Leal‐Costa, Gabriel Segura‐López, Manuel Piñero‐Zapata, and María José Pujalte‐Jesús; investigation, José Luis Díaz‐Agea, Álvaro Ros‐Romero, César Leal‐Costa, Gabriel Segura‐López, Pedro Simón Cayuela‐Fuentes, José Antonio Vera‐Pérez, Juan Manuel Cánovas‐Pallarés, Manuel Piñero‐Zapata, César Cinesi‐Gómez, María Gracia Adánez‐Martínez, and María José Pujalte‐Jesús; methodology, José Luis Díaz‐Agea, Álvaro Ros‐Romero, Gabriel Segura‐López, Pedro Simón Cayuela‐Fuentes, José Antonio Vera‐Pérez, Juan Manuel Cánovas‐Pallarés, and Manuel Piñero‐Zapata; resources, José Luis Díaz‐Agea, José Antonio Vera‐Pérez, César Cinesi‐Gómez, and María Gracia Adánez‐Martínez; supervision, José Luis Díaz‐Agea, and César Leal‐Costa; validation and César Leal‐Costa; writing–original draft, José Luis Díaz‐Agea, and Álvaro Ros‐Romero; writing–review and editing, César Leal‐Costa, Gabriel Segura‐López, Pedro Simón Cayuela‐Fuentes, José Antonio Vera‐Pérez, Juan Manuel Cánovas‐Pallarés, Manuel Piñero‐Zapata, César Cinesi‐Gómez, María Gracia Adánez‐Martínez, and María José Pujalte‐Jesús.

## Funding

This research received no external funding.

## Disclosure

All authors have read and agreed to the published version of the manuscript.

## Ethics Statement

The study was conducted in accordance with the Declaration of Helsinki, and approved Ethics Committee of Catholic University of Murcia (protocol code CE012107 and date of approval 02 February 2021) for studies involving humans.

## Consent

Informed consent was obtained from all subjects involved in the study.

## Conflicts of Interest

The authors declare no conflicts of interest.

## Supporting Information

Table S1. Dimensions and items of the ISCAQ questionnaire.

## Supporting information


**Supporting Information** Additional supporting information can be found online in the Supporting Information section.

## Data Availability

The data that support the findings of this study are available upon request from the corresponding author. The data are not publicly available due to privacy or ethical restrictions.

## References

[1] Amorøe T. N. , Interprofessional Simulation-Based Education and Resilience-Focused Debriefing for Improved Teamwork [Internet], 2024, https://gupea.ub.gu.se/handle/2077/80171.

[2] Xavier N. A. and Brown M. R. , Interprofessional Education in a Simulation Setting, StatPearls [Internet], 2025, StatPearls Publishing, Treasure Island (FL), http://www.ncbi.nlm.nih.gov/books/NBK557471/. 32491403

[3] McCrory K. , Jowsey T. , and Chen Y. , Essential Elements of Preregistration Nursing Interprofessional Simulation Training, Journal of Nursing Education. (2023) 62, no. 1, 28–35, 10.3928/01484834-20221109-02. 36652581

[4] Gough S. , Hellaby M. , Jones N. , and MacKinnon R. , A Review of Undergraduate Interprofessional Simulation-Based Education (IPSE). Collegian, Collegian. (2012) 19, no. 3, 153–170, 10.1016/j.colegn.2012.04.004, 2-s2.0-84865198319. 23101350

[5] Organization W. H. , Framework for Action on Interprofessional Education and Collaborative Practice, 2010, https://iris.who.int/handle/10665/70185. 21174039

[6] Rossler K. , Molloy M. A. , Pastva A. M. , Brown M. , and Xavier N. , Healthcare Simulation Standards of Best PracticeTM Simulation-Enhanced Interprofessional Education, Clinical Simulation in Nursing. (2021) 58, 49–53, 10.1016/j.ecns.2021.08.015.

[7] Watts P. I. , McDermott D. S. , Alinier G. et al., Healthcare Simulation Standards of Best PracticeTM Simulation Design, Clinical Simulation in Nursing. (2021) 58, 14–21, 10.1016/j.ecns.2021.08.009.

[8] Decker S. , Sapp A. , Bibin L. et al., Healthcare Simulation Standards of Best Practice®: The Debriefing Process, Clinical Simulation in Nursing. (2025) https://www.nursingsimulation.org/article/S1876-1399(25)00092-1/fulltext.

[9] Harrison N. , Somerville S. , Kumar P. , and Collins K. , Re-Examining Interprofessional Simulation: Using Social Identity Theory to Explore the Influence of ‘Profession’ on Interprofessional Learning, Clinical Simulation in Nursing. (2024) 97, 10.1016/j.ecns.2024.101652.

[10] Elsayed H. , Nivala M. , and Carlzon L. , Students’ and Instructors’ Perspectives on Learning and Professional Development in the Context of Interprofessional Simulation, Teaching and Learning in Medicine. (2024) 36, no. 4, 454–469, 10.1080/10401334.2023.2230562. 37394980

[11] Olivares S. C. , Moreno N. A. , and Figueroa S. C. , Educación Interprofesional Utilizando La Simulación Clínica Para El Desarrollo De Habilidades Comunicativas, Cogitare Enfermagem. (2025) 30, 10.1590/ce.v30i0.98216es.

[12] Toqan D. , Ayed A. , Khalaf I. A. , and Alsadi M. , Effect of High-Fidelity Simulation on Self-Satisfaction and Self-Confidence Among Nursing Students, SAGE Open Nurs. (2023) 9, 10.1177/23779608231194403. PMC10424544 37584033

[13] Alsabri M. , Boudi Z. , Lauque D. et al., Impact of Teamwork and Communication Training Interventions on Safety Culture and Patient Safety in Emergency Departments: a Systematic Review, Journal of Patient Safety. (2022) 18, no. 1, e351–e361, 10.1097/pts.0000000000000782. 33890752

[14] Flin R. , Winter J. , Sarac C. , and Tomas M. A. R. , Human Factors in Patient Safety: Review of Topics and Tools, 2009, https://abdn.elsevierpure.com/en/publications/human-factors-in-patient-safety-review-of-topics-and-tools/.

[15] Fuchshuber P. and Greif W. , Romanelli J. R. , Dort J. M. , Kowalski R. B. , and Sinha P. , Creating Effective Communication and Teamwork for Patient Safety, The SAGES Manual of Quality, Outcomes and Patient Safety, 2022, Springer International Publishing, Cham, 443–460, https://link.springer.com/10.1007/978-3-030-94610-4_23.

[16] Velasco G. G. , Hernández Gutiérrez L. S. , and Daniel Guerrero A. B. , Escenario De Simulación Clínica Interprofesional Sobre Delirium Mixto En El Pregrado De Medicina Y Fisioterapia, Investig En Educ Médica. (2021) 10, no. 40, 29–36.

[17] Bochatay N. , Ju M. , O’Brien B. C. , and Van Schaik S. M. , A Scoping Review of Interprofessional Simulation-Based Team Training Programs, Simul Healthc J Soc Simul Healthc. (2025) 20, no. 1, 33–41, 10.1097/sih.0000000000000792. PMC11776884 38526045

[18] Krielen P. , Meeuwsen M. , Tan E. C. T. H. , Schieving J. H. , Ruijs A. J. E. M. , and Scherpbier N. D. , Interprofessional Simulation of Acute Care for Nursing and Medical Students: Interprofessional Competencies and Transfer to the Workplace, BMC Medical Education. (2023) 23, no. 1, 10.1186/s12909-023-04053-2. PMC9921059 36774481

[19] Cheng A. , Eppich W. , Kolbe M. , Meguerdichian M. , Bajaj K. , and Grant V. , A Conceptual Framework for the Development of Debriefing Skills: a Journey of Discovery, Growth, and Maturity, Simulation in Healthcare Journal. (2020) 15, no. 1, 55–60, 10.1097/sih.0000000000000398. 31743312

[20] Fenzi G. , Díaz-Agea J. L. , Pethick D. , Bertolín-Delgado R. , Hernández-Donoso N. , and Lorente-Corral L. , An Undergraduate Interprofessional Experience With Self-Learning Methodology in Simulation Environment (MAES©): A Qualitative Study, Nursing Reports. (2022) 12, no. 3, 446–463, 10.3390/nursrep12030043. 35894033 PMC9326708

[21] Hernández E. , Camacho M. , Leal-Costa C. et al., Does Multidisciplinary Team Simulation-Based Training Improve Obstetric Emergencies Skills?, Healthcare. (2021) 9, no. 2. 10.3390/healthcare9020170 PMC7915121 33562857

[22] Díaz-Agea J. L. , Ayensa-Arano C. , Pujlate-Jesus M. J. et al., Improving Interprofessional Team Simulation Learning. One More Step Towards the Humanization of Health Care in Emergency Situations, Signa Vitae. (2021) https://www.signavitae.com/articles/10.22514/sv.2021.223.

[23] Beichler H. , Grandy S. , Neumaier S. , Lilgenau A. , Schwarz H. , and Wagner M. , Interprofessional Paediatric High-Fidelity Simulation Training: A Mixed Methods Study of Experiences and Readiness Among Nursing and Medical Students, Nursing Reports. (2024) 14, no. 1, 566–585, 10.3390/nursrep14010044. 38535716 PMC10974358

[24] Cheng A. , Grant V. , Dieckmann P. , Arora S. , Robinson T. , and Eppich W. , Faculty Development for Simulation Programs: Five Issues for the Future of Debriefing Training, Simulation in Healthcare Journal. (2015) 10, no. 4, 217–222, 10.1097/sih.0000000000000090, 2-s2.0-84939547264. 26098492

[25] Cheng A. , Palaganas J. , Eppich W. , Rudolph J. , Robinson T. , and Grant V. , Co-debriefing for Simulation-based Education: A Primer for Facilitators, Simulation in Healthcare Journal. (2015) 10, no. 2, 69–75, 10.1097/sih.0000000000000077, 2-s2.0-84927935311. 25710318

[26] Kumar P. , Paton C. , Simpson H. M. , King C. M. , and McGowan N. , Is Interprofessional Co-Debriefing Necessary for Effective Interprofessional Learning Within Simulation-Based Education?, International Journal of Healthcare Simulation. (2021) 1, no. 1, 49–55.

[27] Tangpaisarn T. , Phrampus P. E. , and O’Donnell J. M. , Tangpaisarn T. , Phrampus P. E. , and O’Donnell J. M. , Debriefing, Navigating Healthcare Simulation: A Practical Guide for Effective Teaching [Internet], 2025, Springer Nature Switzerland, Cham, 67–77, 10.1007/978-3-031-81265-1_9.

[28] Taylor S. , Landry C. A. , Rachor G. S. , Paluszek M. M. , and Asmundson G. J. G. , Fear and Avoidance of Healthcare Workers: An Important, Under-Recognized Form of Stigmatization During the COVID-19 Pandemic, Journal of Anxiety Disorders. (2020) 75, 10.1016/j.janxdis.2020.102289. PMC7434636 32853884

[29] Taylor S. J. , Bogdan R. , and DeVault M. L. , Introduction to Qualitative Research Methods: A Guidebook and Resource, 2016, 4rd edition, Wiley, Hoboken (N.J.

[30] Elo S. and Kyngäs H. , The Qualitative Content Analysis Process, Journal of Advanced Nursing. (April 2008) 62, no. 1, 107–115, 10.1111/j.1365-2648.2007.04569.x, 2-s2.0-40949147823. 18352969

[31] Braun V. and Clarke V. , Using Thematic Analysis in Psychology, Qualitative Research in Psychology. (January 2006) 3, no. 2, 77–101, 10.1191/1478088706qp063oa, 2-s2.0-33750505977.

[32] SAGE Publications Ltd [Internet], Naturalistic Inquiry. (2025) https://uk.sagepub.com/en-gb/eur/naturalistic-inquiry/book842.

[33] Tong A. , Sainsbury P. , and Craig J. , Consolidated Criteria for Reporting Qualitative Research (COREQ): a 32-item Checklist for Interviews and Focus Groups, International Journal for Quality in Health Care. (2007) 19, no. 6, 349–357, 10.1093/intqhc/mzm042, 2-s2.0-36549063576. 17872937

[34] Labrague L. J. , McEnroe – Petitte D. M. , Fronda D. C. , and Obeidat A. A. , Interprofessional Simulation in Undergraduate Nursing Program: An Integrative Review, Nurse Education Today. (2018) 67, 46–55, 10.1016/j.nedt.2018.05.001, 2-s2.0-85046813792. 29754113

[35] Lee W. , Kim M. , Kang Y. et al., Nursing and Medical Students’ Perceptions of an Interprofessional Simulation-Based Education: A Qualitative Descriptive Study, Korean Journal of Medical Education. (2020) 32, no. 4, 317–327, 10.3946/kjme.2020.179. 33296575 PMC7733731

[36] Margalida M. B. , Práctica Colaborativa Interprofesional En Salud: Conceptos Clave, Factores Y Percepciones De Los Profesionales, Educación Médica. (2018) 1, 21–24.

[37] Teuwen C. , Van Der Burgt S. , Kusurkar R. , Schreurs H. , Daelmans H. , and Peerdeman S. , How Does Interprofessional Education Influence Students’ Perceptions of Collaboration in the Clinical Setting? A Qualitative Study, BMC Medical Education. (2022) 22, no. 1, 10.1186/s12909-022-03372-0. PMC9047320 35477384

[38] Rudolph J. W. , Raemer D. B. , and Simon R. , Establishing a Safe Container for Learning in Simulation: the Role of the Presimulation Briefing, Simulation in Healthcare Journal. (2014) 9, no. 6, 339–349, 10.1097/sih.0000000000000047, 2-s2.0-84919497137. 25188485

[39] Edmondson A. C. , Learning from Failure in Health Care: Frequent Opportunities, Pervasive Barriers, BMJ Quality and Safety. (2004) 13, no. suppl 2, ii3–ii9, 10.1136/qhc.13.suppl_2.ii3. PMC1765808 15576689

[40] Edmondson A. , Psychological Safety and Learning Behavior in Work Teams, Administrative Science Quarterly. (1999) 44, no. 2, 350–383, 10.2307/2666999, 2-s2.0-0033243278.

[41] Webster C. S. , Verstappen A. , Weller J. M. , and Henning M. A. , Translation of Non-Technical Skills and Attitudes to Practice After Undergraduate Interprofessional Simulation Training, Asia Pacific World School. (2025) 10, no. 1, 48–52, 10.29060/taps.2025-10-1/sc3349.

[42] Najjuma J. N. , Muhumuza A. , Santorino D. et al., Barriers and Facilitators to Interprofessional Simulation-Based Learning in a Ugandan Medical School: A Qualitative Study, BMC Medical Education. (2024) 24, no. 1, 10.1186/s12909-024-06521-9. PMC11669217 39722006

[43] Armour T. , Ford R. , and Rasmussen B. , Anaesthetic Nurses’ Perceptions of Learning During Interprofessional Simulation Education, Clinical Simulation in Nursing. (2019) 35, 5–9, 10.1016/j.ecns.2019.06.001, 2-s2.0-85069706095.

[44] Carrera A. M. , Naweed A. , Leigh E. et al., Naweed A. , Wardaszko M. , Leigh E. , and Meijer S. , Constructing Safe Containers for Effective Learning: Vignettes of Breakdown in Psychological Safety During Simulated Scenarios, Intersections in Simulation and Gaming [Internet], 2018, Springer International Publishing, Cham, 15–29, http://link.springer.com/10.1007/978-3-319-78795-4_2.

[45] García-Méndez J. A. , Díaz-Agea J. L. , Leal-Costa C. et al., Simulación Clínica 3.0. El Futuro De La Simulación: El Factor Grupal, Rev Latinoam Simul Clínica. (2022) 4, no. 1, 29–34.

[46] Turner S. and Harder N. , Psychological Safe Environment: A Concept Analysis, Clinical Simulation in Nursing. (2018) 18, 47–55, 10.1016/j.ecns.2018.02.004, 2-s2.0-85044169034.

[47] Díaz Agea J. , Leal Costa C. , García-Méndez J. , Hernández E. , Adánez M. , and Sáez A. , Self-Learning Methodology in Simulated Environments (MAES©): Elements and Characteristics, Clinical Simulation in Nursing. (2016) 12, 268–274.

[48] Chávez-Valenzuela P. , Kappes M. , Sambuceti C. E. , and Díaz-Guio D. A. , Challenges in the Implementation of Inter-professional Education Programs with Clinical Simulation for Health Care Students: A Scoping Review, Nurse Education Today. (2025) 146. 10.1016/j.nedt.2024.106548 39740591

[49] Holmes C. and Mellanby E. , Debriefing Strategies for Interprofessional Simulation—A Qualitative Study, Advanced Theory and Simulations. (2022) 7, no. 1, 10.1186/s41077-022-00214-3. PMC9206121 35717254

[50] Brett-Fleegler M. , Rudolph J. , Eppich W. et al., Debriefing Assessment for Simulation in Healthcare: Development and Psychometric Properties, Simulation in Healthcare Journal. (2012) 7, no. 5, 288–294, 10.1097/sih.0b013e3182620228, 2-s2.0-84867204484. 22902606

