# Supplementary material for: Learners’ Perspectives on Interprofessional Simulation and Co‐Debriefing: An Exploratory Mixed‐Methods Study
Source: J Nurs Manag. 2026 Feb 26;2026:3787497. doi: 10.1155/jonm/3787497 (PMC12937087; doi:10.1155/jonm/3787497)
Supplement: Supplementary file 1 — Supporting Information Additional supporting information can be found online in the Supporting Information section. [file JONM-2026-3787497-s001.docx]

Table S1. Dimensions and items of the ISCAQ questionnaire.

| Applicability: This dimension assesses the opinion on the degree of application and usefulness of the interprofessional training on the real-life practice of health care. Minimum/maximum scores: 3/15 | |
| --- | --- |
| Items | - I believe that interprofessional simulation is beneficial and could help decrease adverse events in the daily practice of healthcare. - Based on my professional experience, I believe that interprofessional simulation is closely aligned with the reality of healthcare. - I have been very interested in the clinical cases planned and solved given their similarity and frequency with which these are found in real health care. |
| Satisfaction with Interprofessional Learning: This dimension assesses participants' satisfaction with the knowledge acquired during interprofessional training. Minim/maximum scores: 2/10 | |
| Items | - My degree of satisfaction with this interprofessional simulation experience is: - I agree that interdisciplinary simulation training such as this should be done in medicine/nursing |
| Motivation: This dimension assesses the degree of motivation reached by the student during the development of interprofessional simulation. Motivation through simulation explains why people initiate, maintain, or discontinue a specific behavior. In this case, if one is motivated, the most probable outcome is that a person will want to participate in more simulation sessions in the future.  Minimum/maximum scores: 3/15 | |
| Items | - I believe that the instructor or facilitator has motivated us sufficiently during the simulated clinical case and its subsequent debriefing. - The clinical cases planned were tailored to the participants' level and were interesting (this encouraged me to become more involved in the case). - I would participate in this learning experience again if given the opportunity. |
| Safe Environment: This dimension refers to the comfort and trust experienced during the simulation training sessions.  Minimum/maximum score: 5/25 | |
| Items | - During the simulation, I felt I was in a safe and respectful environment, where both disciplines were just as important. - Group dynamics were performed before the development of the clinical cases to facilitate respectful learning and harmony in the team. - I felt comfortable during the discussion of the cases, and I believe that my opinion was respected. - I perceived that what was important was the learning, and I did not feel evaluated. - The facilitator/s have worried about creating a psychologically safe environment in the group. |
| Organization: This dimension assesses students' perceptions of the facilitators' participation in the design and development of clinical cases.  Minimum/maximum scores: 3:15 | |
| Items | - I believe that the facilitators have organized the sessions adequately, considering the training needs of both disciplines. - I believe that the sessions and clinical cases have been previously prepared by the facilitators in a thorough and adequate manner. - I believe that the learning objectives were met during the interprofessional simulation, thanks to the facilitators' mediation during the debriefing. |
| Co-Debriefing: The main characteristics perceived about the Debriefing are assessed when it was conducted by two facilitators.  Minimum/maximum scores: 7/35 | |
| Items | - The co-debriefers were well-adjusted and conducted the session in a manner that was harmonious and characterized by a good understanding between them. - I believe that co-debriefing has given equal importance to both disciplines (nursing and medicine). - I believe that co-debriefing has had a positive influence on learning due to the diverse opinions and experiences of the facilitators. - I believe that the co-debriefers have clearly stated the learning objectives and the participants’ roles, which has facilitated the better development of the case and the debriefing. - I believe that a good relationship and effective communication existed between the facilitators, which has positively influenced the session. - I believe that the presence of two facilitators (one from each profession) has helped resolve the group's conflicts, should they have arisen. - The co-debriefers did not step on each other, and interference or tension was not found with respect to any of the subjects discussed. The co-debriefers complemented each other and intervened only when necessary in the pre-established subjects or spheres assigned to each of them. |
